# Examining the Role of Buzzing Time and Acoustics on Pollen Extraction of *Solanum elaeagnifolium*

**DOI:** 10.3390/plants10122592

**Published:** 2021-11-26

**Authors:** Mandeep Tayal, Rupesh Kariyat

**Affiliations:** 1 Department of Plant and Environmental Sciences, Clemson University, Clemson, SC 29634, USA; mtayal@clemson.edu; 2 Department of Biology, The University of Texas Rio Grande Valley, Edinburg, TX 78539, USA; 3 School of Earth, Environment and Marine Sciences, The University of Texas Rio Grande Valley, Edinburg, TX 78539, USA

**Keywords:** buzz pollination, weeds, poricidal anthers, buzzing frequency, amplitude

## Abstract

Buzz pollination is a specialized pollination syndrome that requires vibrational energy to extract concealed pollen grains from poricidal anthers. Although a large body of work has examined the ecology of buzz pollination, whether acoustic properties of buzz pollinators affect pollen extraction is less understood, especially in weeds and invasive species. We examined the pollination biology of Silverleaf nightshade (*Solanum elaeagnifolium*), a worldwide invasive weed, in its native range in the Lower Rio Grande Valley (LRGV) in south Texas. Over two years, we documented the floral visitors on *S. elaeagnifolium*, their acoustic parameters (buzzing amplitude, frequency, and duration of buzzing) and estimated the effects of the latter two factors on pollen extraction. We found five major bee genera: *Exomalopsis, Halictus, Megachile, Bombus*, and *Xylocopa*, as the most common floral visitors on *S. elaeagnifolium* in the LRGV. Bee genera varied in their duration of total buzzing time, duration of each visit, and mass. While we did not find any significant differences in buzzing frequency among different genera, an artificial pollen collection experiment using an electric toothbrush showed that the amount of pollen extracted is significantly affected by the duration of buzzing. We conclude that regardless of buzzing frequency, buzzing duration is the most critical factor in pollen removal in this species.

## 1. Introduction

Buzz pollination, a specialized pollination syndrome, is found in ~6% of flowering plants, where pollen grains are concealed inside poricidal anthers [1,2,3,4]. It is suggested that the buzz pollinated plants are a typical example of convergent evolution, as similar flower morphologies appear to evolve among different unrelated families. For instance, *Solanum-*type flowers have evolved across Primilaceae, Gesneriaceae, and Ericaceae, in addition to Solanaceae with typical characteristics of buzz-pollinated species. These include the presence of poricidal anthers and the lack of nectar or other pollen rewards, which dispense pollen only to the authorized buzz pollinators [2,5,6,7]. Flowers from most of the *Solanum* spp. are exclusively buzz pollinated that control the rate of pollen removal as well as exclude pollen thieves such as hoverflies (*Simosyrphus spp*.) and stingless bees (*Trigona spp*.) [6,8,9,10,11]. Buzz pollination also provides us an excellent model system to understand the origin of complex floral modifications, the evolutionary ecology of pollen rewards, as well as biomechanics underlying plant-pollinator interactions [3,6,12,13,14]. Although a large body of previous work focused on understanding the role of plant and pollinator characteristics in the context of buzz pollination in various plant families, whether such interactions play a key role in the reproductive success of invasive species such as *S. elaeagnifolium* is less understood.

Even though buzz pollination is known for more than 100 years [15,16], only recently, the field has received a rejuvenated interest [12,13,17,18]. In the past decade, exceptional work has been done on understanding buzz pollination in different plant and bee species, their evolutionary and ecological consequences, as well as both plant and pollinator fitness [3,6,12,13,17,19,20,21,22,23,24]. For example, although bee vibrational acceleration (instantaneous changes in amplitude, ms^−2^) and frequency (number of cycles per second, Hz) are independent of bee mass and flower mass and vary between bee species, floral characteristics have been found to significantly affect their transmission to the flowers [3]. Moreover, the vibrations produced by the bumble bee (*Bombus* spp.), a major buzzing pollinator, with greater amplitude and longer duration, ejects more pollen in any given foraging effort [25]. Collectively, these studies demonstrate that bee and floral traits can affect acoustic properties of bees during buzz pollination [3]. 

Although previous studies documented the morphological and acoustic characteristics of buzzing bees and their host plants, we are still at the early stages of understanding such species-specific interactions at the community level [14,26,27,28,29]. This is more ecologically relevant in invasive weeds, where pollination success, and consequently seed set, is a driving force in invasion success. For instance, a comparison of *S. elaeagnifolium* populations within and beyond their ancestral range, Petanidou et al. (2018) found variations in resource allocation patterns that directly affect the pollinator visitation rate and fruit set [28]. Moreover, being self-incompatible and having nectar-less flowers, understanding the intricate details of the pollination biology of such weed species is important, as their reproduction ability, and hence propagule supply, plays a critical role in their uncontrollable spread and establishment [30]. To examine this, we documented the pollination biology of *S. elaeagnifolium* in the LRGV by identifying the major buzz pollinators, their buzzing acoustic parameters (buzzing frequency, amplitude, duration of buzzing), as well estimating the effects of frequency and duration of buzzing on pollen extraction. Following this, we then used electric toothbrushes [14] to simulate the natural buzzing frequency and duration of buzzing to validate the results from the native pollinators. 

We hypothesized that buzz pollinators will vary in their buzzing properties (frequency, amplitude) during *S. elaeagnifolium* pollination and consequently aid in seed set. Apart from threatening the pollination success of other native plants [26,30], *S. elaeagnifolium* also serves as a reservoir host for ‘*Candidatus* Liberibacter solanacearum’ which causes Zebra Chip disease of the potato [31]. Understanding the role of *S. elaeagnifolium* pollinators and studying the effects of buzz pollination traits such as buzzing frequency on *S. elaeagnifolium* reproductive success in the native range will provide us better knowledge of how pollination traits affect the invasiveness of the species.

Specifically, we asked two major questions. 1) Who are the major buzz pollinators of *S. elaeagnifolium* in their native range, and 2) How do variations in bee buzzing traits affect pollen extraction? To answer these questions, we used a combination of field and lab studies over two years across the Lower Rio Grande Valley, Texas, USA, the native range of *S. elaeagnifolium*. 

## 2. Results

### 2.1. Major Pollinators of S. elaeagnifolium in Its Native Range in LRGV, Texas

**Figure 1:**
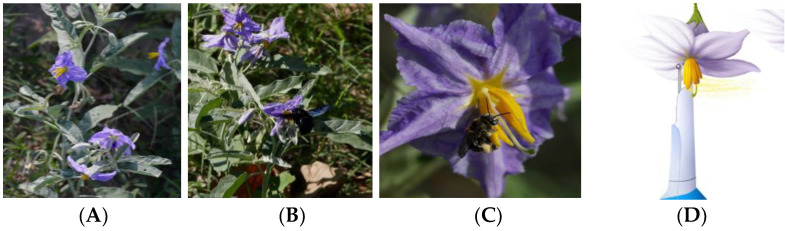
Silverleaf nightshade (SLN; *Solanum elaeagnifolium*) flowers and pollinators in its native range in south Texas. (**A**) Inflorescence, (**B**) *Xylocopa* spp., and (**C**) *Exomalopsis* spp. buzz pollinating SLN, and (**D**) artificial pollination using electric toothbrush (modified from [14]).

Bee specimens collected in field surveys were directly examined and visualized under a dissecting microscope. Upon identification, we found five different bee genera prevalent in the Lower Rio Grande Valley of Texas. These include (A) *Exomalopsis*, (B) *Halictus*, (C) *Megachile*, (D) *Bombus*, and (E) *Xylocopa* spp. We also identified a subset of the bees to the species level and found that they were predominantly *Xylocopa mexicanorum*, *Halictus ligatus*, *Bombus pennsylvanicus*, and *Exomalopsis mellipes*. The *Bombus* and *Xylocopa* are significantly bigger bees, while *Exomalopsis*, *Halictus*, and *Megachile* bees are comparatively smaller in size (Figure 1). The taxonomic confirmation of each collected bee to the species level would require additional resources and expertise and was not carried out for this study. The specimens are however stored for further analyses and DNA barcoding.

### 2.2. Bee Visit Time

**Figure 2:**
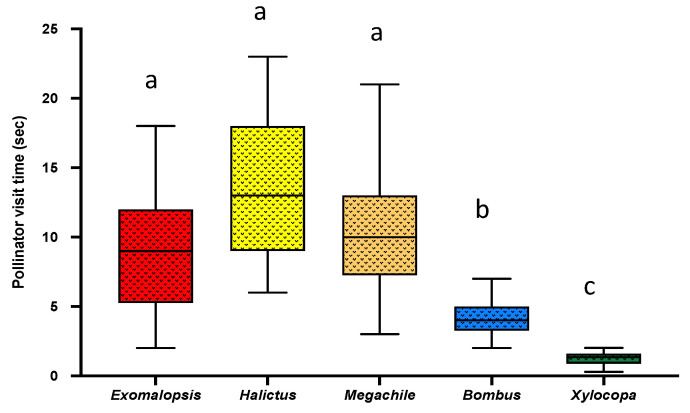
Box and whisker plot of the results of One-Way ANOVA and post-hoc Tukey’s HSD test of comparison of bee visit time (N = 40) among major five genera of buzz pollinating bees in LRGV, Texas. Different letters show significant differences among means (*p* < 0.05).

Bee visitation time (N = 40, Table 1) (the total time (seconds) spent by each bee on each flower) was estimated as the difference between when the bee landed on the flower and when the bee left the flower. Among all five genera, we found that *Xylocopa*, the largest bee genera surveyed, spent the lowest amount of time (Mean = 1.24 ± SE = 0.66; One-Way ANOVA; *p* < 0.0001) followed by the *Bombus* bee which spent significantly longer than *Xylocopa* (Mean = 4.05 ± SE = 0.81; *p* < 0.0001) but less than other small bees (*Exomalopsis*: Mean = 9.08 ± SE = 0.69; *p* = 0.0001, *Megachile*: Mean = 10.05 ± SE = 0.14; *p* = 0.0018, *Halictus*: Mean = 13.73 ± SE = 0.07; *p* = 0.0008 (Figure 2). The larger the bee mass/size, the higher the buzzing amplitude (i.e., the function of the physical condition of the bee), which gives an advantage to bee genera for efficient pollen extraction in less time [25]. However, there was no significant difference in bee visit time among *Exomalopsis*, *Halictus*, and *Megachile* (Figure 2), which also had comparable body mass as shown in Section 2.6. 

### 2.3. No. of Pulses Per Bee Visit and Buzz % over Visit Time

**Figure 3:**
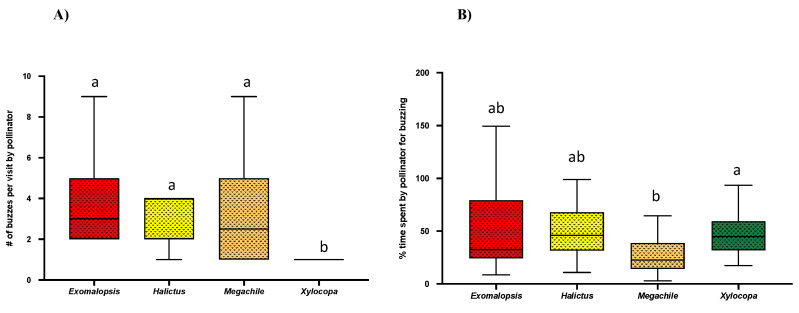
Box and whisker plots of the results of Kruskal–Wallis, One-way ANOVA, and post-hoc Tukey’s HSD (*p* < 0.05), for comparison (**A**) No. of buzzes/visit and (**B**) Buzz % over visit time among different bee genera. Different letters show significant differences among means (*p* < 0.05).

The analysis of bee vibrations using Audacity software showed that each bee visit included multiple pulses (bee buzzing) and time lags (bee not buzzing) within a single visit. After counting pulses/buzz among different genera, we found that *Xylocopa* (N = 22) produces significantly fewer pulses ~1 per buzz (Mean = 1 ± SE = 0; Kruskal–Wallis test; *p* < 0.001) as compared to other genera. However, we did not see any significant difference in the number of pulses/buzzes among *Exomalopsis* (N = 18), *Halictus* (N = 8), and *Megachile* (N = 16) genera (Figure 3A, Table 1). While estimating the number of pulses/buzzes in a single flower visit, we also measured the duration of each pulse, and the pooled pulse time was recorded as actual buzz time per flower visit. We calculated buzz % over visit time using the ratio of actual buzz time over total visit time. Interestingly, we found that *Megachile* had a significantly lower buzz % (Mean = 16.8 ± SE = 4.2) over the visit time as compared to *Xylocopa* (Mean = 27.45 ± SE = 4.20; One-way ANOVA; *p* < 0.0158), while there were no significant differences in buzz % over visit time among *Exomaplosis*, *Halictus*, and *Xylocopa* (Figure 3B).

### 2.4. Bee Buzzing Frequency and Buzzing Amplitude

**Figure 4:**
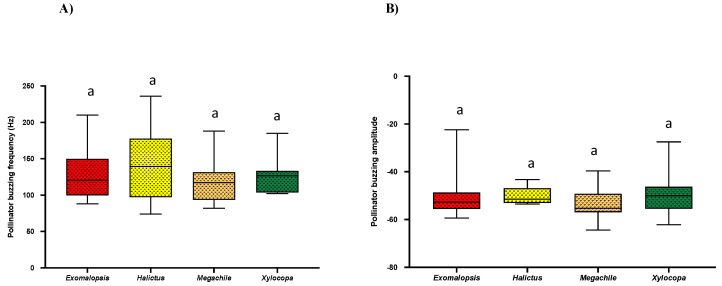
Box and whisker plots of the results of non-parametric, Kruskal–Wallis test (*p* < 0.05) and post-hoc Dunn’s test for comparison of (**A**) Bee buzzing frequency and (**B**) Buzzing amplitude among different bee genera. Similar letters show non-significant differences (*p* < 0.05).

Buzzing frequency (Hz) is the number of vibrations produced per second, while buzzing amplitude (dB) denotes how loud the vibrations are produced during buzzing. On vibrational analysis, we did not detect any significant differences in bee buzzing frequency (*Exomalopsis*: Mean = 126 ± SE = 29.68, *Halictus*: Mean = 144 ± SE = 50.73, *Megachile*: Mean = 119 ± SE = 29.66, and *Xylocopa*: Mean = 125 ± SE = 26.71; Kruskal–Wallis test; *p* = 0.4519) and buzzing amplitude (*Exomalopsis*: Mean = −50.6 ± SE = −11.9, *Halictus*: Mean = −50.1 ± SE = −17.7, *Megachile*: Mean = −53.8 ± SE = −13.44, and *Xylocopa*: Mean = −50.2 ± SE = −10.7; Kruskal–Wallis test; *p* = 0.3018) among all genera (Figure 4A,B). However, *Bombus* was not included in such analyses because we were unable to record their buzzing vibrations.

### 2.5. Acoustics of First and Last Buzz

**Figure 5:**
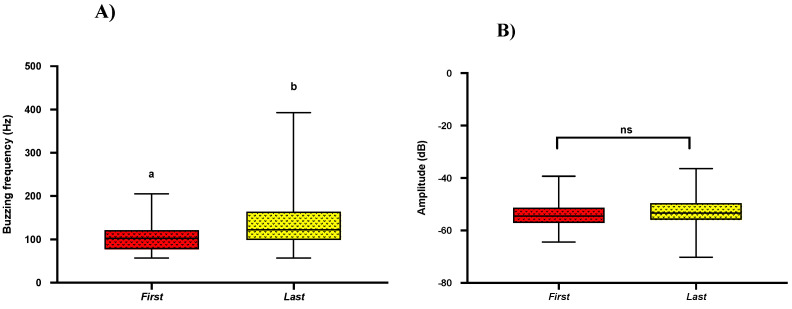

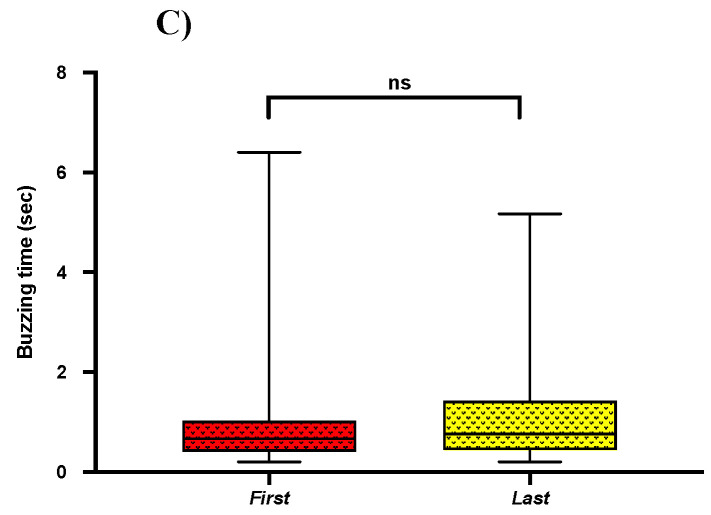
Box and whisker plots of the results of *t*-test (*p* < 0.05) for comparison of (**A**) Buzzing frequency, (**B**) Buzzing amplitude, and (**C**) Buzzing time in between first and last buzz. Similar letters show the non-significant differences. Different letters show significant differences (*p* < 0.05).

Previous studies show that bees extract most of their pollen (~60%) in the first two pulses [2,25]. To find if there are any variations among buzzes, we compared buzzing acoustic parameters (frequency, amplitude, buzzing time) of the first and last pulse. In our pairwise comparisons, regardless of the bee genera, we found that bees produced the first pulse with a significantly lower frequency when compared to the last pulse frequency (Mean = 110 ± SE = 5.93; Mean = 136 ± SE = 9.24; *t*-test; *p* = 0.021) (Figure 5A). However, we found no significant differences in buzzing amplitude (Mean = −53.75 ± SE = −8.29; Mean = −53.04 ± SE = −8.18; *t*-test; *p* = 0.54) and buzzing time (Mean = 1.16 ± SE = 0.18; Mean = 1.16 ± SE = 0.18; *t*-test; *p* = 0.99) between the first and last buzz (Figure 5B,C).

### 2.6. Comparison of Bee Size among Different Genera

**Figure 6:**
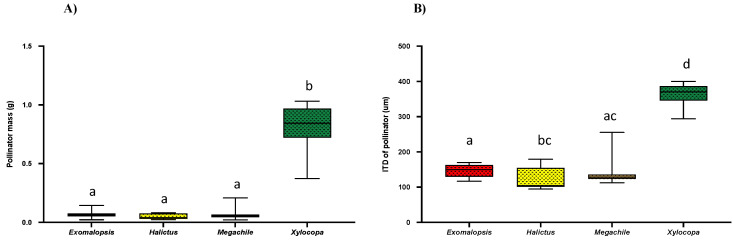
Box and whisker plots of the results of non-parametric, Kruskal–Wallis test (*p* < 0.05) and post-hoc Dunn’s test for comparison of bee size; (**A**) Bee mass and (**B**) ITD. Different letters show significant differences among means (*p* < 0.05).

As bee size (bee mass and ITD: the distance between a bee’s wing bases or tegulae) is a key factor in determining bee buzzing acoustics (frequency, amplitude) [3,13,18,32,33,34], we compared the average bee mass (g) and ITD of all four genera (Figure 6A,B). We found that *Xylocopa* had a significant higher mass (Mean = 0.836 ± 0.18; Kruskal–Wallis test; *p* < 0.0001) as compared to other genera, while there was no significant difference among *Exomalopsis* (Mean = 0.066 ± 0.015), *Halictus* (Mean = 0.048 ± 0.017), and *Megachile* (Mean = 0.067 ± 0.017) (Figure 6A). Moreover, similar trends were observed for ITDs with having a significant higher ITD of *Xylocopa* (Mean = 365.9 ± 78; Kruskal–Wallis test; *p* < 0.0001), while there was insignificant difference among *Exomalopsis* (Mean = 146.33 ± 34.5), *Halictus* (Mean = 124.32 ± 43.95), and *Megachile* (Mean = 143.65 ± 35.91) (Figure 6B). 

### 2.7. Artificial Pollen Extraction and Buzzing Time Intervals

**Figure 7:**
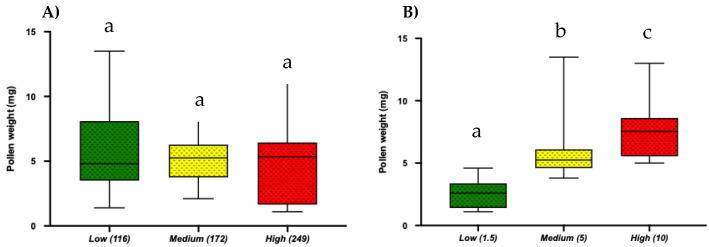
Box and whisker plots of the results of One-way ANOVA, Tukey’s HSD (*p* < 0.05) for the effect of (**A**) Buzzing frequency and (**B**) Buzzing time (Low: 1.5 s, Medium: 5 s, High: 10 s) on artificial pollen extraction. Different letters show significant differences among means (*p* < 0.05).

Using electric toothbrushes in the lab, we examined if different frequency levels and buzzing time intervals affected the amount of pollen collected. We found that the amount of pollen collected was independent of buzzing frequency. On the other hand, buzzing time had a significant effect on the amount of pollen collected with longer buzzing time (10 s) resulting in more pollen extraction (Mean = 7.73 ± SE = 0.45; One-way ANOVA; *p* < 0.0001), while shorter buzzing time (~1.5 s) extracted a significantly lower amount of pollen (Mean = 2.61 ± SE = 0.22; One-way ANOVA; *p* < 0.0001; Figure 7A,B). Clearly, the length of buzzing time is a critical factor for efficient pollen collection.

## 3. Discussion

Our study provides valuable information on major floral visitors of *S. elaeagnifolium* as well as variation in their visit time and buzzing acoustics in their native range. As *S. elaeagnifolium* is a noxious and worldwide invasive weed threatening natural and agricultural ecosystems due to their high propagule supply [28], our study characterizing the time and acoustic parameters of buzzing bees will be of interest to understand the role and efficiency of pollinators in the invasion success of this species. In the field survey study, we found five major genera of buzz-pollinating bees: *Exomalopsis*, *Halictus*, *Megachile*, *Bombus,* and *Xylocopa* as the most prevalent in the LRGV of Texas. *Exomalopsis*, *Xylocopa,* and *Bombus* are members of the Apidae family, while *Halictus* and *Megachile* fall in Halictidae and Megachilidae, respectively. The *Xylocopa* and *Bombus* are comparatively bigger bees, while *Exomalopsis*, *Halictus,* and *Megachile* are smaller [13]. Previous studies demonstrated that these bees play a key role in pollinating other buzz-pollinated plants such as *S. rostratum* as well despite their size variations [11]. Interestingly, the absence of honeybees (*Apis melifera*) in *S. elaeagnifolium* visits shows how restricted pollen release is important, and only specialized pollinators can extract pollen, as pollen larceny is costly for plants [7,11,25]. The evolution of hereanthery (stamen dimorphism within angiosperms, lack of floral nectaries and poricidal anthers) within buzz pollination is possibly such a response to pollen thieves aimed at reducing pollen consumption by them [35]. 

Investigating how bee visit time varies among different genera, we found that small bees, i.e., *Halictus*, *Exomalopsis,* and *Megachile*, spent a significantly longer time than larger bees, i.e., *Bombus* and *Xylocopa* (Figure 2). This could be due to the competitive disadvantage of small bees due to their inability to generate vibrations at a higher magnitude when compared to larger bees. Consequently, they compensate it by spending a longer time on each flower to extract enough pollen in any given foraging effort [13,34,36]. The longer the time spent by a bee on each flower, anthers are stimulated with force longer; hence, more pollen is extracted [25]. Moreover, within each buzz, we found multiple pulses and time lags, a characteristic feature of a typical buzz (Figure 3A) [1,34]. Interestingly, among all bees, *Megachile* bees were found to have the lowest buzz % over visit time when compared to other genera although they visited each flower longer (Figure 3B), a possible effect of small bees trying to minimize their energy investment and extract more pollen in any given foraging effort. 

In the recent past, more work has documented the effects of bee traits (size, species) on buzzing efficiency [3,6,12,17,18,37]. However, we lack any documentation of such characteristics in *S. elaeagnifolium* in its native range in south Texas. Over two years of experiments, we were able to compare the buzzing frequency and amplitude of four bee genera: *Exomalopsis*, *Halictus*, *Megachile,* and *Xylocopa* (*Bombus* was not included in such analyses because we were unable to record their buzzing vibrations), and surprisingly, we could not find any significant differences in buzzing frequency and buzzing amplitude among them (Figure 4A,B). Previous studies show that bees tend to extract maximum pollen (~60%) in the first and second pulses while the rest of the pulses only contribute to ~19% [2]. To study if such interactions prevail in *S. elaeagnifolium* pollination, we compared buzzing frequency, buzzing amplitude, and duration of buzzing time of the first and last pulses for all bees. We found a significant difference in buzzing frequency in the first and last pulses of visiting bees having a significantly lower frequency of the first pulse, as lower frequency (~124 Hz) is close to plant stamen frequency, which could result in better vibration transmission and, consequently, efficient pollen extraction (Figure 5A) [2]. It has been well documented that insect body size can influence flight and floral vibration frequencies and amplitude either negatively or positively [12,19,25,36]. Comparing bee size (bee mass and ITD) among different genera, the significantly heavier *Xylocopa* would have an advantage for efficient pollen collection with minimum foraging efforts [12,25].

To understand the effect of buzzing frequency and duration of buzzing time on the amount of pollen removed further [14], we ran an artificial pollen extraction experiment, which clearly demonstrated that pollen extraction is independent of buzzing frequency (Figure 7A), while the duration of buzzing time has a significant effect (Figure 7B). These findings also agree with [25], where they found that a longer duration of buzzes eject more pollen in *S. rostratum*, while frequency had no significant effect on the amount of pollen removed. As discussed before, small bees spend more time to extract pollen as compared to bigger bees, in which the latter can extract a similar amount of pollen in less time in any given foraging effort [13,17,25,38,39,40]. 

To conclude, using field-based insect-visitation surveys, acoustics assessment, and toothbrush-based artificial pollen extraction experiments, we conclude that the duration of buzzing time is the most critical factor in *S. elaeagnifolium* pollen extraction. We also show that although there are variations in bee size and their vibrational buzzing parameters, *S. elaeagnifolium* is visited by multiple bee genera in its native range and consequently results in a high seed set and propagule supply [30]. Future studies should be focused on fruit and/or seed-set to estimate the efficacy of pollination by the observed insect visitors experimentally, an area we are currently exploring. The comparison of *S. elaeagnifolium*-pollinator interactions in natural and managed agricultural systems would be useful for developing better management strategies. 

## 4. Materials and Methods

### 4.1. Study System

*S. elaeagnifolium* is a noxious weed, native to the Southern United States and Northern Mexico while invasive in other continents [26,28,30,41]. It has blue-to-lilac hermaphrodite nectar-less flowers with poricidal anthers, which offers pollen as a reward to the buzz pollinators. *S. elaeagnifolium* propagate by seeds, dispersed by wind, water, birds, and grazing animals, and asexually through rhizomes [42,43,44] (Figure 1A). Previous studies show that *S. elaeagnifolium* is visited by various pollinators belonging to Apidae (*Xylocopa spp*, *Bombus* spp.), Halictidae (*Nomina* spp. and *Pseudapis* spp.), as well small bees of genus *Megachile* [28].

### 4.2. Field Survey and Bee Incidence

In this study, we surveyed silverleaf nightshade (*S. elaeagnifolium*) sub-populations across the Lower Rio Grande Valley, Texas, USA. In the Spring–Summer (April–August) of 2019 and 2020, we selected four field plots: (1) PPC Farms, City of Mission, TX, USA (26°16′84.25″N 98°31′45.47″W), (2) Edinburg, Texas, USA (26°20′18.1″N 98°11′17.5″W), (3) San Juan, Texas, USA (26°11′24.9″N 98°08′51.1″W), 4) Mission, Texas, USA (26°10′33.5″N 98°18′48.7″W), all within a 50-mile radius, as our study locations for pollinator assessment. *S. elaeagnifolium* populations were observed in small patches of various sizes measured in the area (m^2^) (PPC farm field: 100 m^2^, Edinburg: 60 m^2^, San Juan: 321 m^2^, Mission: 400 m^2^). Multiple field trips were made to observe major buzz pollinator incidence and record bee buzzing vibrations. Field visits were made early in the morning (7 a.m.–9 a.m.) to record maximum pollinator activity, and to confirm that we document first visitors once the flowers have opened [14]. During each visit, bee data were collected for their flower visit (number of visits), the time between landing and leaving a flower (visit bout), while simultaneously recording bee buzzes (explained in Section 4.3) followed by capturing a subset of bees for identification, bee mass and Intertegular distance (ITD) measurements carried out in the lab. Time spent between bee landing and bee leaving the flower was recorded as flower visit time. For each field visit, two researchers worked as a team to collect the data for 3 h/visit to minimize any recording errors.

### 4.3. Bee Vibrations Recording and Bee Capturing

When the bee buzzes, floral sonication is produced as a by-product of vibrations emitted from the bee exoskeleton transmitted to the anthers [1]. Previous studies established that floral sonication can be characterized by analyzing acoustic measures of duration and fundamental frequency [13]. To record and characterize floral sonication (produced in an audible sound), bees were observed in the early morning during their flower visits. Once the bee landed on the flower, a digital audio recorder Tascam DR-100 MK-III (TEAC America, Inc., Montebello, CA, USA) was held within 1–5 cm of the bee with the pointing microphone head towards the bee’s dorsal side until the bee left the flower, and these recordings were saved as wave files (.wav) (Sampling frequency = 10 recordings/h). After recording bee buzzing vibrations, bees were captured using an insect net and transferred to the 50 mL centrifuge tubes. Each tube had 50% ethanol dipped cotton balls, and each bee was later identified and labeled for mass and ITD measurements.

### 4.4. Estimating Vibration Frequency and Amplitude

Vibrational frequency and amplitude are characteristic perimeters of floral sonication vibrations. We used a freely accessible Audacity v. 2.1.3 (https://sourceforge.net/projects/audacity/, accessed on 23 September 2019) software to calculate bee vibrational frequency (Hertz or Hz), duration of vibration (s), and vibration amplitude (dB) [13]. Before analysis, recordings were listened to twice to identify extraneous noise, including wind, birds, and machinery. Then, this noise was filtered out (noise reduction = 12 dB, sensitivity = 6, frequency smoothing = 3) from a small portion of each recording, followed by a batch process filtration from the whole recording. Peak frequency/fundamental frequency (lowest frequency in the vibration with the largest amplitude) was calculated by using the “Plot Spectrum” function (FFT = 8192 Hz, Hamming window). Each vibration was analyzed by examining a spectrogram of the recording using the “Spectrogram” function (FFT = 8192 Hz, hamming window). Fundamental frequency and amplitude of a given vibration were determined with corresponding peak frequency with the largest amplitude in a spectrogram [20]. Duration of vibration was also calculated in Audacity by selecting the bee buzzing area in the spectrogram.

### 4.5. Bee Mass and Intertegular Distance (ITD)

Bee body mass and Intertegular distance (ITD) are reliable parameters of bee size characteristics [13]. All captured bees were stored in the refrigerator (4℃) for bee identification and body measurements (bee mass and ITD). Bee mass (g) was calculated by weighing each bee individually on an advanced digital balance (Accuris Series Dx, Model: W3101A-220, Benchmark Scientific, NJ, USA). For ITD measurements, each bee was observed under a compound microscope at 10X magnification. ITD (µm) was measured as the distance between the bee’s wing tegulae across the thoracic dorsum. After body mass and ITD measurements, each bee was placed back in corresponding tubes and stored at 4 °C.

### 4.6. Artificial Pollen Extraction

In addition to field surveys, a lab experiment was conducted to estimate the effect of different vibrational frequencies and duration of buzzing time on the amount of pollen extracted. To do so in the lab, we selected electric toothbrushes [14]; Figure 1D), which had vibrational frequency ranges (137 Hz–249 Hz), and the duration of toothbrushes use was determined based on data recorded from the field. We collected newly opened virgin flowers from the field early in the morning (to avoid any pollination by bees) and brought them to the lab. For pollen extraction, we used electric toothbrushes of different strokes, i.e., 14,000/min (Oral-B 3d White Action Power Toothbrush), 20,000/min (Colgate 360 powered toothbrush, Colgate Co. Pvt. Ltd.), and 30,000/min (Vivid Sonic Clean toothbrush) having three frequency levels (137 Hz, 173 Hz, and 249 Hz: [14]), respectively, for three buzzing times (1.5 s, 5 s, and 10 s) in a full factorial design. Collected pollen was weighed (in mg) and recorded for each treatment.

#### Statistical Analysis

All the collected data were checked for their distribution (normal or not), followed by parametric or non-parametric tests for analyses. Due to the non-normal nature of bee frequency, bee amplitude, bee mass, and ITD, data were analyzed using the Kruskal–Wallis test (*p* < 0.05). However, as bee visit time, bee buzz % of visit time followed a normal distribution, we used One-Way ANOVA followed by pairwise comparisons using Tukey’s HSD test (*p* < 0.05). On the other hand, the bee acoustic differences between the first and last buzz were analyzed using an unpaired *t*-test (*p* < 0.05). For artificial pollen extraction data, we used One-Way ANOVA followed by Tukey’s HSD (*p* < 0.05) to analyze the effect of frequency and length of buzzing time on the amount of pollen extracted. All data were analyzed in statistical software JMP (SAS Institute, Cary, NC, USA) and GraphPad PRISM (La Jolla, CA, USA).

## Figures and Tables

**Table 1:** Number of individuals (N) recorded for bee acoustic parameters.

Bee Genera/Parameters	*Exomalopsis*	*Halictus*	*Megachile*	*Xylocopa*
Bee visitation time	40	40	40	40
Number of pulses/visits	18	8	16	22
Buzz % over time	18	8	16	22
Bee Buzzing frequency and amplitude	18	8	16	22
Bee size (Bee mass and ITD)	18	8	16	22

## Data Availability

Data collected for this study are available from Dryad Digital Repository.
